# Carotid Sinus Tumor-Induced Positional Bradycardia and Hypotension After Extubation: A Case Report

**DOI:** 10.7759/cureus.54013

**Published:** 2024-02-11

**Authors:** Colin Kirsch, Areen Badwal, Romain Rabany, Julia Shabanian, Carla Dormer

**Affiliations:** 1 Anesthesiology, Creighton University School of Medicine, Phoenix, USA; 2 Psychiatry, Creighton University School of Medicine, Phoenix, USA; 3 Anesthesiology, Valleywise Health Medical Center, Phoenix, USA; 4 Anesthesiology, Creighton University Arizona Health Education Alliance, Phoenix, USA; 5 Anesthesiology, District Medical Group, Phoenix, USA

**Keywords:** vasovagal response, carotid sinus hypersensitivity, hemodynamic instability, carotid sinus compression, malignancy compression, posture-dependent symptoms, bradycardia, intraoperative hypotension, head and neck cancer, carotid sinus syndrome (css)

## Abstract

Regional progression of head and neck malignancies can lead to carotid sinus tumors, causing hemodynamic instability and carotid sinus syndrome (CSS). A 60-year-old male with tonsillar squamous cell carcinoma developed profound positional bradycardia and hypotension immediately after extubation following dental extraction. The patient developed recurrent episodes of positional bradycardia and hypotension, leading to eventual pacemaker placement. Further workup revealed a large mass in the left neck and necrotic cervical lymphadenopathy, indicating CSS from malignancy compression. This case highlights the need for consideration of CSS in patients with known head and neck malignancy, particularly when postural hypotension and bradycardia are present.

## Introduction

Regional progression of head and neck malignancies can lead to carotid sinus compression, causing hemodynamic instability and carotid sinus syndrome (CSS). CSS can be caused by compression or irritation of the carotid sinus by tumors or masses in the head and neck region, leading to cardiovascular symptoms, such as bradycardia, hypotension, syncope, and dizziness, often triggered by changes in the head position or carotid sinus stimulation [1,2]. It is an important phenomenon to recognize in surgical patients, as mechanical compression and anesthesia can exacerbate vasodepressor reflexes. We present an in-depth case analysis of a patient with advanced head and neck cancer who developed profound intraoperative hypotension and bradycardia concerning underlying CSS.

This article was previously presented as a poster abstract at the 2023 American Society of Anesthesiologists (ASA) Annual Meeting on October 15, 2023.

## Case presentation

A 60-year-old homeless male with a past medical history of alcohol and methamphetamine abuse presented with untreated, advanced-stage head and neck squamous cell carcinoma for a planned dental extraction procedure to treat severe dental caries before initiation of chemotherapy and radiation. His head and neck cancer was first diagnosed after presenting to the emergency department with a two-month history of progressive sore throat, odynophagia, and perceived difficulty with swallowing. Workup at the time included a CT neck, which showed a 2.7 cm left posterior-lateral tongue mass and multiple pathologically enlarged cervical lymph nodes concerning metastasis. The largest abnormal lymph node measured 2.2 cm in diameter on the right and 1.4 cm on the left. A larger lymph node conglomerate was likely present on the left, but it was difficult to characterize on the CT.

Additional scattered cervical lymphadenopathy was noted, left greater than right. He underwent a biopsy, which confirmed poorly differentiated squamous cell carcinoma of the left tonsil with lymph node involvement, deemed clinical stage T2N1M0. Positron emission tomography-computed tomography (PET-CT) demonstrated hypermetabolic activity within the bilateral tonsils and multiple hypermetabolic lymph nodes in the left neck, indicating a suspicious cancer spread. The patient had largely refused active cancer treatment over the following year due to social and substance use barriers. His disease course was complicated by malnutrition, with a BMI of 18.02. Surgical history was non-contributory. Outpatient medications included tamsulosin and over-the-counter ranitidine.

**Figure 1:**
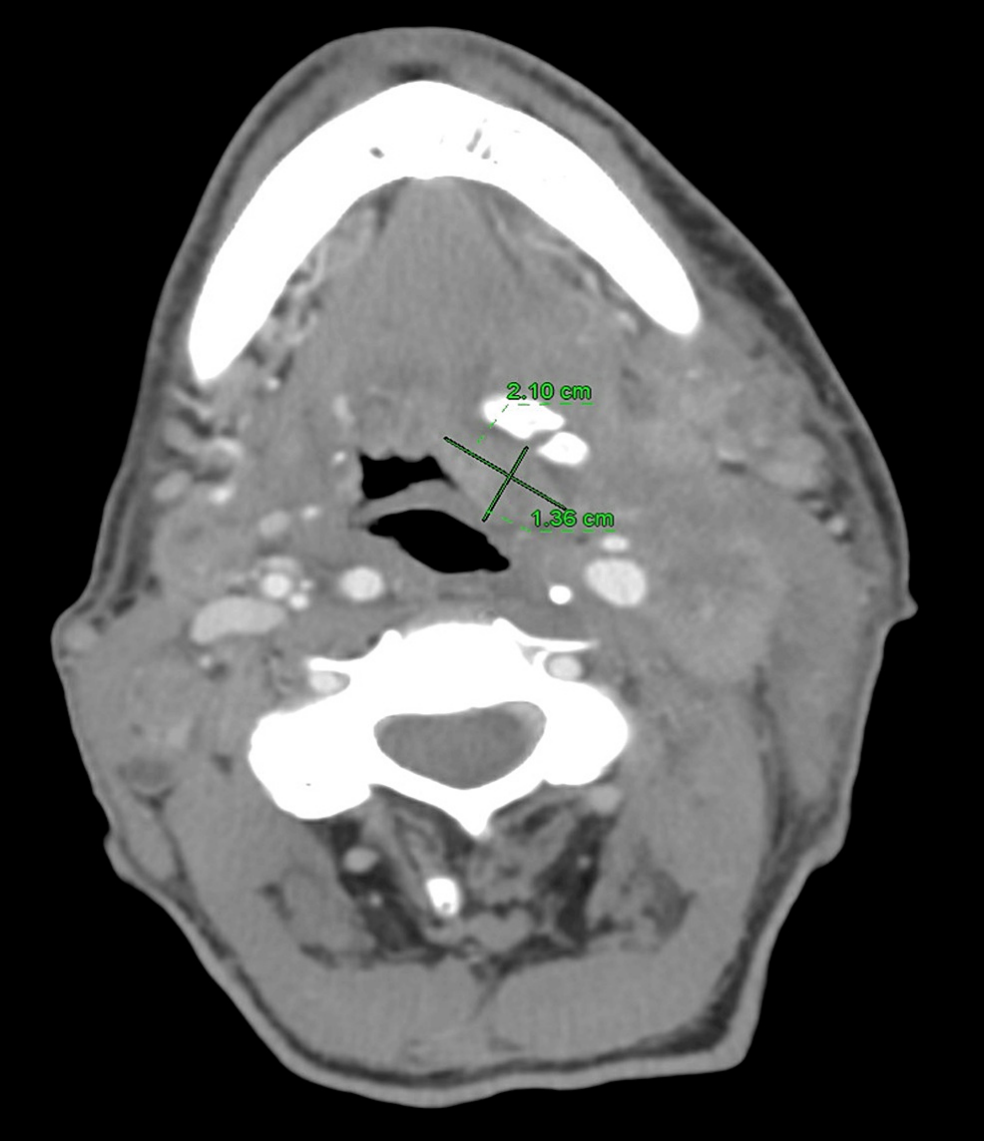
CT soft tissue neck with IV contrast Imaging demonstrates an ill-defined soft tissue mass within the left hypopharynx and extending into the left pre-epiglottic vallecula. This is indistinct. However, it measures approximately 5 cm x 2.2 cm x 1.7 cm (green). Diffuse bulky necrotic cervical lymphadenopathy is noted, most consistent with squamous cell carcinoma.

The patient presented to the operating room for the scheduled extraction of damaged dentition before cancer therapy. Preoperative laboratory tests were significant only for mild normocytic anemia and hypoproteinemia; the remainder of his complete blood count, comprehensive metabolic panel, and coagulation panel were within normal limits. Preoperative ECG showed sinus bradycardia at 59; otherwise, it was a normal ECG. An MRI of the soft tissues of the neck with and without contrast had been obtained, showing the progression of a known left-sided head and neck malignancy with diffuse necrosis and enlargement of cervical lymph nodes bilaterally. The malignancy measured up to 5.8 cm at its greatest dimensions, representing a near-complete obstruction of his aerodigestive tract (Figure 1).

**Figure 2:**
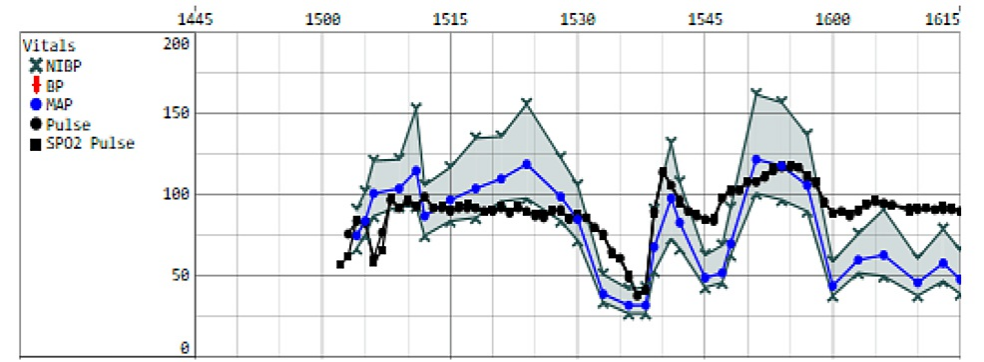
Intraoperative record of the patient's hemodynamics The image portrays the patient variable hemodynamics intraoperatively. Epinephrine and phenylephrine were given between 1535 and 1540.

The patient's vitals were stable before the procedure with a blood pressure of 139/78, pulse of 78, respirations of 16, temperature of 98.2˚F, and SpO_2_ of 96%. Standard ASA monitors were placed alongside IV and arterial access before commencing the procedure. The patient was induced under general anesthesia with 70 mg lidocaine, 125 mcg fentanyl, 100 mg succinylcholine, 30 mg rocuronium, and 120 mg IV propofol and maintained on sevoflurane gas anesthetics. Intubation was uneventful. The dental extraction lasted approximately 25 minutes and occurred without apparent complications. Within half an hour of case induction, the anesthesiology team noted the patient's blood pressure falling precipitously with a systolic at 50 mmHg, declining to a nadir of 47 mmHg at its lowest. This was met with spikes in heart rate initially into the 140s, followed by a rapid onset of profound bradycardia, with rates dropping from 90 beats per minute to 33 over the next few minutes. The anesthesia team administered several doses of phenylephrine and epinephrine with good response, temporarily improving his mean arterial pressures into the 70s and 80s and heart rate to 91 bpm (Figure 2).

Unfortunately, upon emergence and extubation, the patient again became severely hypotensive and bradycardic almost immediately after elevating the head of his hospital bed to 30 degrees. Repeat measurements showed mean arterial pressures (MAPs) in the very low 50s down to 36 mmHg and heart rates in the 30s with one reading as low as 34 bpm. At this point, the primary anesthesia attending decided to abort the planned additional general surgery procedure of laparoscopic G-tube placement and initiate resuscitative efforts.

A bedside cardiac ultrasound was performed, which showed new evidence of hypovolemia from decreased right atrial filling. Intravenous fluid boluses with 2 liters of normal saline were administered, temporarily improving his blood pressure but requiring repeat dosing of vasoactive medications for support. The patient was transferred directly to the surgical intensive care unit for further monitoring and treatment, given the precarious nature of his hemodynamic status. The patient continued to demonstrate episodic hypotension and bradycardia overnight on postoperative day zero. These were described as posture-dependent in nature, improving when supine but dropping significantly upon sitting up or changing position. Morning labs were obtained that were grossly unremarkable, arguing against anemia, electrolyte disturbance, or poor nutrition accounting for the spells.

The cardiology team was consulted to evaluate the concern for intrinsic heart block or dysfunction versus extrinsic compression at the level of the carotid sinus structures. Their leading hypothesis after a review of available imaging and the patient's history was a diagnosis of CSS manifesting acutely in the operative setting. By postoperative day two, the patient underwent successful pacemaker placement for chronic bradycardic support. Vasoactive medications, including midodrine and intermittent phenylephrine, were also initiated for afterload support after the patient had additional breakthrough hypotensive episodes with position changes despite cardiac pacing.

The medicine team decided to proceed with the scheduled laparoscopic feeding tube placement for nutritional optimization on postoperative day nine before the patient started cancer therapy. Interestingly, they noted difficulties in perioperatively managing the patient's blood pressure during the procedure. Several boluses of intravenous fluids were required throughout to counteract drops in pressure with stimulation, similar to the initial presentation. Tube feeds were started cautiously at trophic rates and uptitrated as tolerated across the remaining hospital stay. With oral intake remaining limited from the tumor bulk, nutrition was managed using the Dobhoff tube until chemotherapy and radiation could hopefully reduce the disease burden.

The patient was eventually transferred out of the ICU on hospital day 14 once repeat episodes of orthostasis had been controlled with the above interventions. He underwent physical and occupational therapy evaluation and was cleared from a functional status standpoint with only minor lingering generalized weakness that was non-focal. No skilled nursing needs were anticipated at the time of discharge. The patient was instructed to establish outpatient care for ongoing cancer treatment and chronic disease management.

## Discussion

**Figure 3:**
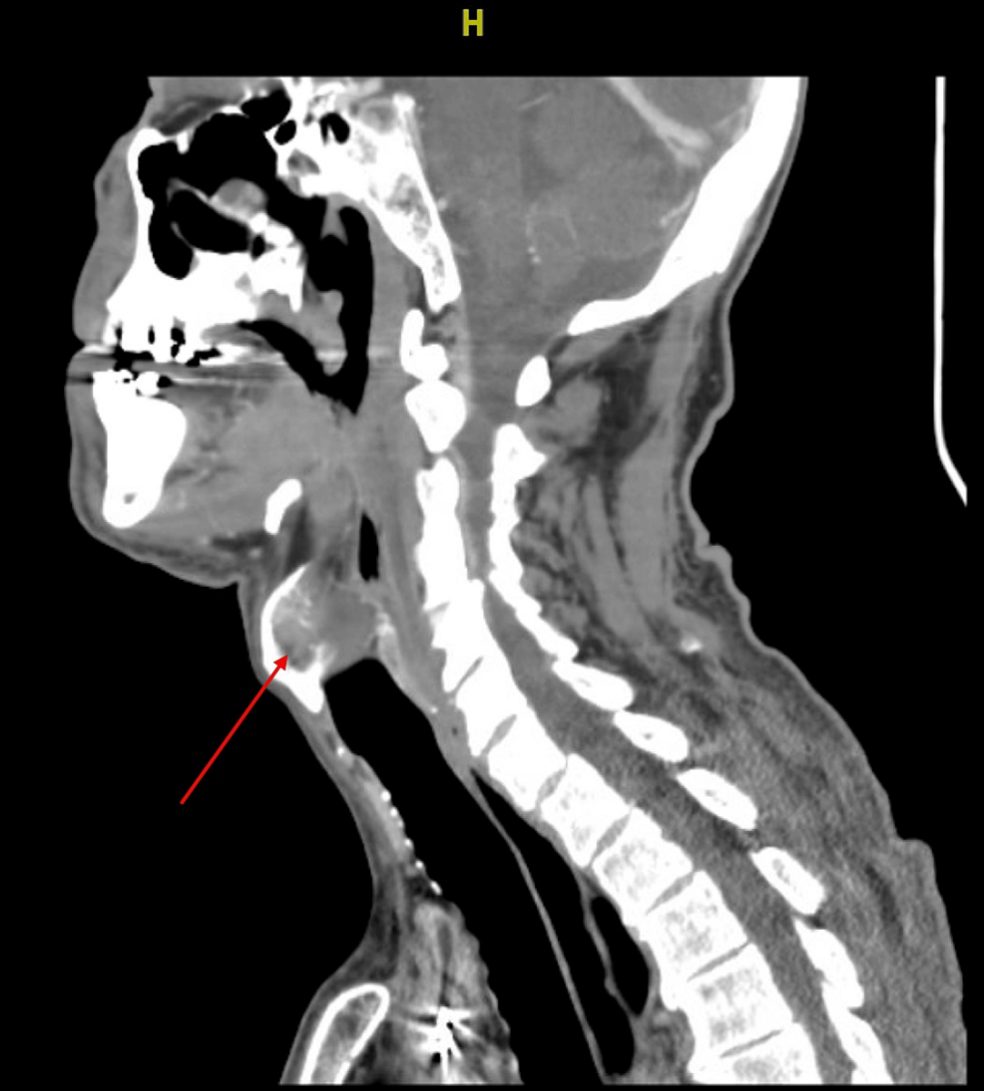
Sagittal CT of the head and neck This figure shows how the left hypopharynx extends into the left pre-epiglottic vallecula where there is an ill-defined soft tissue mass (red arrow). There is diffuse bulky centrally necrotic conglomerate lymphadenopathy throughout the bilateral cervical chains.

This case first illustrates the initial diagnostic challenge of new-onset intraoperative hemodynamic instability with head and neck cancer, where the differential remains broad. Hypovolemia, cardiac dysfunction, vasovagal response, distributive shock from infection or adrenal insufficiency, and medication reactions can all present similarly and were considered actively by the clinical teams involved [3]. The acute, episodic, posture-dependent symptoms raised suspicion for extrinsic cardiovascular compression, malignant invasion of the glossopharyngeal and vagus nerves, and mechanical distortion of baroreceptor pathways were appropriately identified as likely etiologies. Local tumor effects were visible on recent MRI imaging, showing diffuse lymphadenopathy and mass effect in the cervical region (Figure 3).

Critically, however, extrinsic compression at the level of the carotid sinus structures by neck malignancy, known as CSS, appears to be the most consistent unifying explanation behind this patient's presyncope and fluctuating hemodynamics. The carotid sinus, located at the bifurcation of the internal and external carotid arteries, functions as one of the body's peripheral arterial baroreceptors involved in moment-to-moment blood pressure regulation [4]. Under normal conditions, stretching of the sinus triggers reflex tachycardia and vasoconstriction to counteract falling pressures [4]. The afferent signal pathway involves the glossopharyngeal nerve, which relays to the vasomotor center, while efferent responses occur via the vagus nerve, which controls cardiac function [5]. In CSS, distortion of the carotid sinus anatomy by external mass effect leads to inappropriate activation of this pathway. Rather than correcting hypotension, mechanical stimulation causes paradoxical bradycardia and peripheral vasodilation that worsens shock [4]. This phenomenon classically manifests as episodic hypotension and slowed heart rates precipitated by neck turning, shaving, tight collars, or direct sinus palpation [6]. From a mechanistic standpoint, both the glossopharyngeal and vagus cranial nerves were likely impinged in this patient at baseline from extensive cervical metastasis visible on MRI (Figures 1, 3). This left him prone to hemodynamic spells that were then exacerbated by standard anesthesia induction protocols involving hyperextension of the neck and repeated neck repositioning. The transient improvement in blood pressure with fluids also aligns with exaggerated baroreflex behavior.

Notably, while pacemaker placement is recommended to help control contributory bradycardia, the root dysfunction behind CSS remains the loss of peripheral vascular resistance from aberrant vasodilation [7]. As such, chronotropic support alone frequently provides incomplete treatment. Medications to promote vasoconstriction, including midodrine, an alpha-agonist, are better-directed therapy for breakthrough hypotension. Medications to promote vasoconstriction, including midodrine, an alpha-agonist, are better-directed therapy for breakthrough hypotension once bradycardia is controlled [8]. This likely explains why the patient had improved hemodynamic stability only after the initiation of pressor support in addition to cardiac pacing.

Moreover, the difficulty noted during subsequent anesthesia for his feeding tube placement provides corroborative evidence of an underlying carotid sinus hypersensitivity. Despite pacemaker management, he continued demonstrating episodic intraoperative hypotension consistent with residual baroreflex disorder. Ongoing vigilance under sedation and surgical stimulation was required.

## Conclusions

This case demonstrates CSS as a secondary complication of advanced cervical cancer leading to intraoperative and postoperative hemodynamic instability. The patient’s known head and neck cancer with a recent demonstration of diffuse local progression provides mechanistic plausibility behind acute CSS manifestation. The stereotypical posture-dependent symptoms and treatment response to fluids and chronotropes initially, followed by pressors later, offer diagnostic clarity once pacing was established. Ultimately, a multidisciplinary approach via tumor-directed therapy, physiologic support through cardiac pacing, and pharmacological optimization targeting vascular tone offered reasonable management when definitive resolution was not possible. It serves as an essential example highlighting carotid sinus hypersensitivity as an anesthesia and perioperative consideration in head and neck cancer.
